# Caring for the caregivers: breast and cervical cancer screening among informal caregivers of cancer patients – a scoping review

**DOI:** 10.1186/s12875-026-03333-2

**Published:** 2026-05-04

**Authors:** Anusree Prabhakaran, Edlin Glane Mathias, Rithu Sathiyamoorthy, Nishit Kumar Bebarta, Shirley Lewis, Arathi P. Rao

**Affiliations:** 1 Department of Global Public Health Policy and Governance, Prasanna School of Public Health, Manipal Academy of Higher Education, Manipal, India; 2 Department of Health Technology and Informatics, Prasanna School of Public Health, Manipal Academy of Higher Education, Manipal, India; 3 Department of Radiation Oncology, Kasturba Medical College, Manipal Academy of Higher Education, Manipal, India

**Keywords:** Breast cancer, Cervical cancer, Cancer screening, Informal caregivers, Sustainable development goal 3

## Abstract

**Background:**

Breast and cervical cancer remain major contributors to the global morbidity and mortality among women, with early detection through screening being the key to prevention. Multiple factors influence the screening uptake, and informal caregiving for a family member with cancer may further influence their engagement in cancer screening. Evidence on breast and cervical cancer screening among women who are informal cancer caregivers remains fragmented. Hence, this scoping review aimed to identify the factors influencing and interventions that enhanced breast and cervical cancer screening, among women who are informal caregivers of cancer patients.

**Methods:**

Following the JBI methodology for scoping review, a comprehensive search was conducted in PubMed, CINAHL, Web of Science, Embase, Scopus, and grey literature sources. A predefined set of inclusion and exclusion criteria was used to select the studies from across the globe. Data was extracted and narratively synthesised, with supporting tables and diagrams.

**Results:**

Nine studies from Asia, Africa, Europe and North America were included. Though some studies reported that caregiving status was linked to greater uptake of breast and cervical cancer screening, lack of knowledge and fear of cancer diagnosis hindered screening for both cancers. Breast cancer screening was higher among older, educated caregivers and female relatives (e.g., sisters), and was facilitated by positive outcome expectations, goal setting, perceived self-efficacy, and social support. Fear of cancer recurrence presented a nuanced association; moderate fear enhanced age-appropriate breast cancer screening, while higher fear led to excessive screening. Cervical cancer screening was hindered by fear of pain, social consequences, cultural beliefs, stigma, privacy concerns, limited access to screening centres, and husbands’ hesitancy. A telephonic counselling by a trained nurse enhanced breast cancer screening; no interventions were found that enhanced cervical cancer screening in informal cancer caregivers, indicating a significant research gap.

**Conclusion:**

Most identified factors mirrored those reported among the general female population, with limited exploration of caregiving-specific contextual factors, and caregiver-specific interventions for breast and cervical cancer screening remain scarce. Integrating physician-initiated education and counselling, and opportunistic screening during the routine clinical encounters for the cancer patient presents a promising approach to enhance breast and cervical cancer screening among informal cancer caregivers.

**Supplementary Information:**

The online version contains supplementary material available at 10.1186/s12875-026-03333-2.

## Introduction

With more than 2 million new cases and 0.6 million deaths, breast cancer is the most commonly diagnosed cancer and with more than 0.6 million new cases and 0.3 million deaths, cervical cancer is the fourth-most commonly diagnosed cancer among women globally [1]. Early detection through screening is crucial for the prevention of these cancers [2]. Increasing women’s participation in breast and cervical cancer screening is therefore a public health priority within the cancer prevention strategies.

Evidence from countries across the world suggests that women’s participation in cancer screening is influenced by the interplay of multiple individual, socio-cultural, health-system and structural factors. Lack of knowledge, perceived risk, privacy concerns, embarrassment, support from spouse and family, culture, religious belief, distance to the screening facility and cost of screening were reported as common influencing factors from lower and middle-income countries [3, 4]. Similarly, lack of knowledge, lack of an invitation for screening, fear of an abnormal test result, and difficulty in remembering appointments were the common factors reported from high-income countries [5, 6].

In addition to these factors, family responsibilities are a crucial determinant of their cancer screening practices. Findings indicate that women’s role as wives or mothers often compelled them to prioritise the family’s needs and other household responsibilities over self-care and undergoing breast and cervical cancer screening [7]. In this context, informal caregiving constitutes an additional layer of responsibility. It involves providing unpaid care and support to an individual with a chronic condition and is often performed by a spouse, relative, friend, or neighbour who shares a close personal relationship with the patient [8].

Informal caregiving for family members with cancer is particularly demanding as it involves a wide range of support, including medical, physical, psychological, and financial assistance to the patients [9]. Evidence indicates that informal caregivers of cancer patients face diverse challenges, including caregiving burden [10], poor sleep [11], stress, and poor quality of life [12]. Moreover, it is evident that once a family member is diagnosed with cancer, any financial resources of the family will be prioritised to provide care to the patient, leaving less attention to other apparently healthy individuals like informal caregivers [13]. Evidence on the impact of cancer diagnosis on the health behaviour of caregivers remains inconclusive, with mixed findings; a few suggesting caregiving may promote health-protective behaviour and others suggesting health-deleterious behaviour among cancer caregivers [14]. It is understood that they often sacrifice time for social and leisure activities and devote minimal attention to their health and well-being, including failing to undergo health checkups [15, 16].

The World Health Organisation estimates that one in five individuals is expected to be diagnosed with cancer in their lifetime [17]. This rise in cancer leads to more individuals assuming the role of informal caregivers, and women constitute the majority [18, 19]. Existing research on informal caregivers of cancer patients largely focuses on their psychological outcomes and caregiving roles, with limited attention to their own preventive health practices [20–22]. In order to address the rising burden of breast and cervical cancer, cancer screening should be prioritised for these caregivers, just as prioritizing treatment for the cancer patients. Tailored strategies addressing barriers unique to caregivers are therefore important. However, the existing studies on breast and cervical cancer screening largely focus on women in general, with limited attention to these caregivers [3–6, 23–27]. Though they have equal opportunity for screening as the general female population, there will be other contextual factors related to the cancer diagnosis in the family and caregiving responsibilities which can influence their screening decisions.

Hence, this topic was chosen for the review with the aim of comprehensively mapping the existing evidence on factors influencing screening uptake and interventions that enhanced it among women who are informal caregivers of cancer patients. These insights can inform researchers in developing a contextually appropriate intervention for enhancing cancer screening in this population, which has received limited research attention. This review was a preliminary work as part of a primary study aimed at developing and assessing an intervention to enhance cancer screening among informal cancer caregivers.

We conducted a preliminary PubMed search, which indicated that the available literature on this topic is limited and heterogeneous in design and in the outcomes assessed. This variability limits the feasibility of conducting a systematic review or meta-analysis. Also, our aim was to identify existing interventions, not to assess their effectiveness. Therefore, a scoping review methodology was considered most appropriate, as it is a rigorous, well-established approach for comprehensively mapping existing evidence and identifying gaps in the literature to guide future research.

## Methods

### Protocol and registration

This scoping review was conducted in accordance with the Joanna Briggs Institute (JBI) guidelines for scoping reviews [28], informed by the scoping review framework proposed by “Arksey and O’Malley” in 2005, which involves five stages: Identifying the research question, identifying relevant studies, study selection, charting the data and collating, summarizing and reporting the results [29]. The review is reported following the “Preferred Reporting Items for Systematic Reviews and Meta Analyses guidelines for scoping reviews (PRISMA-ScR)” [30]. The PRISMA-ScR checklist is attached as Supplementary File 1. The protocol of this scoping review is registered in the “Open Science Framework” (10.17605/OSF.IO/2BZRF).

### Stage 1: identifying the research question

This scoping review was conducted to answer the following research questions.

1. What are the factors influencing the uptake of breast and cervical cancer screening among women who are informal caregivers of cancer patients?
2. What are the interventions that enhanced the uptake of breast and cervical cancer screening among women who are informal caregivers of cancer patients?

### Stage 2: identifying relevant studies

To identify all relevant articles, this scoping review followed a three-step search strategy. Firstly, an initial search was conducted in MEDLINE (PubMed) and Google Scholar. Key articles were identified through this search, and the keywords and MeSH terms from their titles and abstracts were used to develop a comprehensive search string. Subsequently, a comprehensive search string was developed in PubMed using a combination of keywords: “informal caregivers”, “family caregivers”, “breast cancer”, “cervical cancer”, and “cancer screening”, connected with the Boolean operators AND/OR. The full search string is provided in Supplementary File 2. This search string was translated for other databases using the Polyglot Search Translator [31]. The search was conducted across the following electronic databases on 2nd December 2025: PubMed (NCBI), CINAHL (EBSCO), Web of Science (Clarivate), Embase (Elsevier) and Cochrane Library. Additionally, grey literature was searched on Google Scholar, Google, Lens.org, WHO ICTRP, the Health Technology Assessment Database and Episteminikos. For Google Scholar, we screened the first 10 results pages, sorted by relevance. Potentially relevant studies were identified through title and snippet screening and saved using the “Save” feature. These records were later exported and uploaded to Rayyan for deduplication and title/abstract screening. Similarly, for Google, we screened the first 10 results pages and downloaded potentially relevant studies for further title/abstract and full-text screening. Finally, the reference list of all included studies was examined to identify any relevant studies that were not captured during the initial two searches. Studies published in the English language from inception were considered for the review. The search was updated before submission of the manuscript, and no new studies relevant to the research questions were identified.

### Stage 3: study selection

The identified articles from each database were imported into the Rayyan software, and duplicates were removed [32]. Three independent reviewers (AP, RS, NKB) performed the title/abstract and full-text screening of the articles according to the inclusion and exclusion criteria presented in Table 1, and any conflicts were resolved by discussion with a fourth reviewer (APR).

**Table 1:** Criteria for inclusion and exclusion based on the Population Concept Context (PCC) framework

	Inclusion criteria	Exclusion criteria
Population	1) Studies were included if they were conducted among informal caregivers of individuals diagnosed with or who survived cancer. Informal caregivers are individuals who have a personal relationship with the cancer patient, such as family members, close relatives, or friends, providing unpaid care and assistance (informal care) to the patient [19] 2) Studies were also included if they were conducted among individuals who live with the cancer patient or have lived with them for at least one year [33, 34] 3) Since this scoping review focuses on breast and cervical cancer screening, studies conducted among female caregivers were primarily included. However, studies that included both male and female caregivers were also considered eligible, but only data pertaining to female caregivers were extracted. In such cases, studies were included if they reported outcomes specific to breast and/or cervical cancer screening, as these correspond to female caregivers.	1.) Studies conducted among relatives/first-degree relatives without specific mention of their caregiving role were excluded.
Concept	1) Factors influencing breast and/or cervical cancer screening 2) Interventions enhancing breast and/or cervical cancer screening	
Context	Global	

Initially, our inclusion criteria were restricted to studies conducted among informal caregivers who were identified as the primary/main caregivers of cancer patients. However, this criterion yielded a very limited number of eligible studies. To broaden the evidence base, we expanded our inclusion criteria to include studies conducted among individuals who live or have lived with cancer patients for at least 1 year. This one-year threshold was informed by two prior studies identified during the search [33, 34]. While co-residence does not always guarantee active caregiving, this criterion was included primarily to capture additional data on potential screening behaviours among individuals closely associated with cancer patients, while acknowledging the lower certainty regarding their caregiving involvement.

In studies including both male and female participants, we assessed whether an outcome specific to breast and/or cervical cancer screening was reported, as these correspond to female caregivers. Studies lacking this were intended to be excluded; however, no such studies were encountered.

In the remaining section of the manuscript, women who are informal caregivers of cancer patients will be referred to as “informal cancer caregivers” for the ease of reading.

Since this review was conducted as a scoping review aimed at identifying and mapping existing evidence on factors and interventions (rather than assessing the effectiveness of the interventions), and in line with the JBI guidelines for conducting scoping reviews (which we followed for our review process), critical appraisal/quality assessment is generally not considered a mandatory step for scoping reviews [28]; hence, a critical appraisal/quality assessment of included studies was not performed. Also, since we had limited interventions and our aim was not to assess the effectiveness of the intervention, but rather to identify the existing interventions, the Risk of bias assessment was not performed.

### Stage 4: charting the data

Data from each study was extracted into a data extraction sheet developed by the authors in Microsoft Excel. The data extracted from each study included details on (a) citation, (b) objective of the study, (c) study design, (d) study setting, (e)sample size, (f) type of cancer screening included (breast or cervical cancer), (g) inclusion criteria of participants (definition of caregiver) (h) caregivers’ relationship to the patient, (i) whether the caregiver is living with the patient or not, (j) whether caregiving burden assessed/not in the study, (k) diagnosis of the patient, (l) stage of cancer, (m) duration of cancer diagnosis, (n) whether the patient is in inpatient or outpatient treatment, (o) factors influencing the uptake of breast as well as cervical cancer screening (p) details of the interventions such as type of intervention, comparator, follow-up interval, mode of intervention delivery, frequency of intervention, duration of intervention and description of the intervention. A draft of the data extraction sheet was developed and pilot-tested by entering details of a few studies to ensure it captured all information relevant to the research question, and it was further refined to improve clarity and comprehensiveness. AP, RS and NKB independently carried out the data extraction, and EGM validated the data extraction sheet.

### Stage 5: collating, summarising, and reporting the results

Results were first summarised in tables, followed by a narrative synthesis with supporting pictorial representations.

## Results

### Overview of study inclusion

**Fig. 1:**
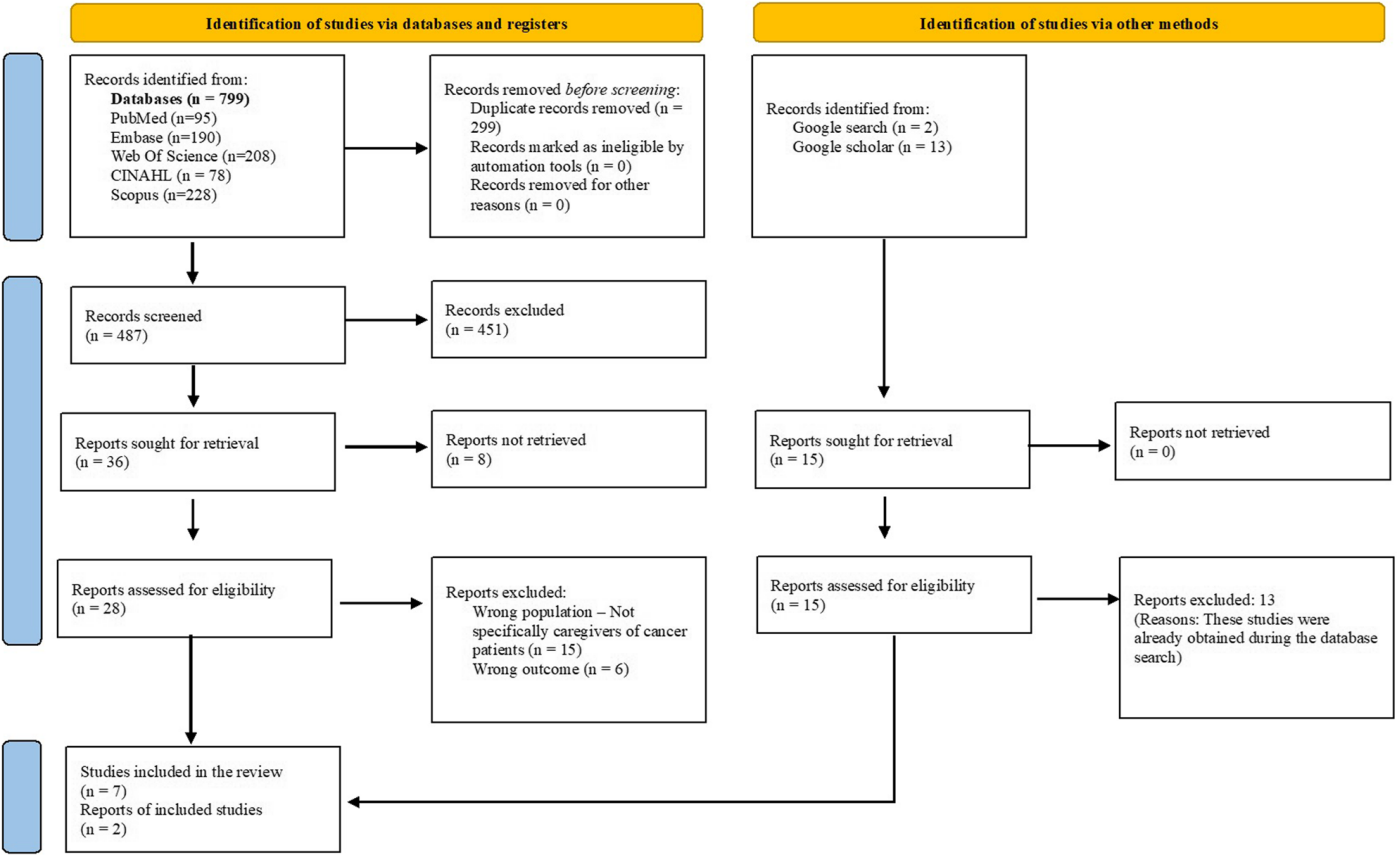
PRISMA flow diagram showing the selection of studies

The search yielded 814 studies across the databases and grey literature sources mentioned above. After removing duplicate studies, three independent reviewers (AP, RS, and NKB) conducted title/abstract and full-text screening. After full-text screening, nine studies met the inclusion criteria. The final included studies were obtained from PubMed, Web of Science, and through a Google search. Citation search didn’t yield any new studies. The search results and the detailed process of study inclusion are depicted in the PRISMA flowchart [35, 36] in Fig. 1.

### Characteristics of the included studies

**Fig. 2:**
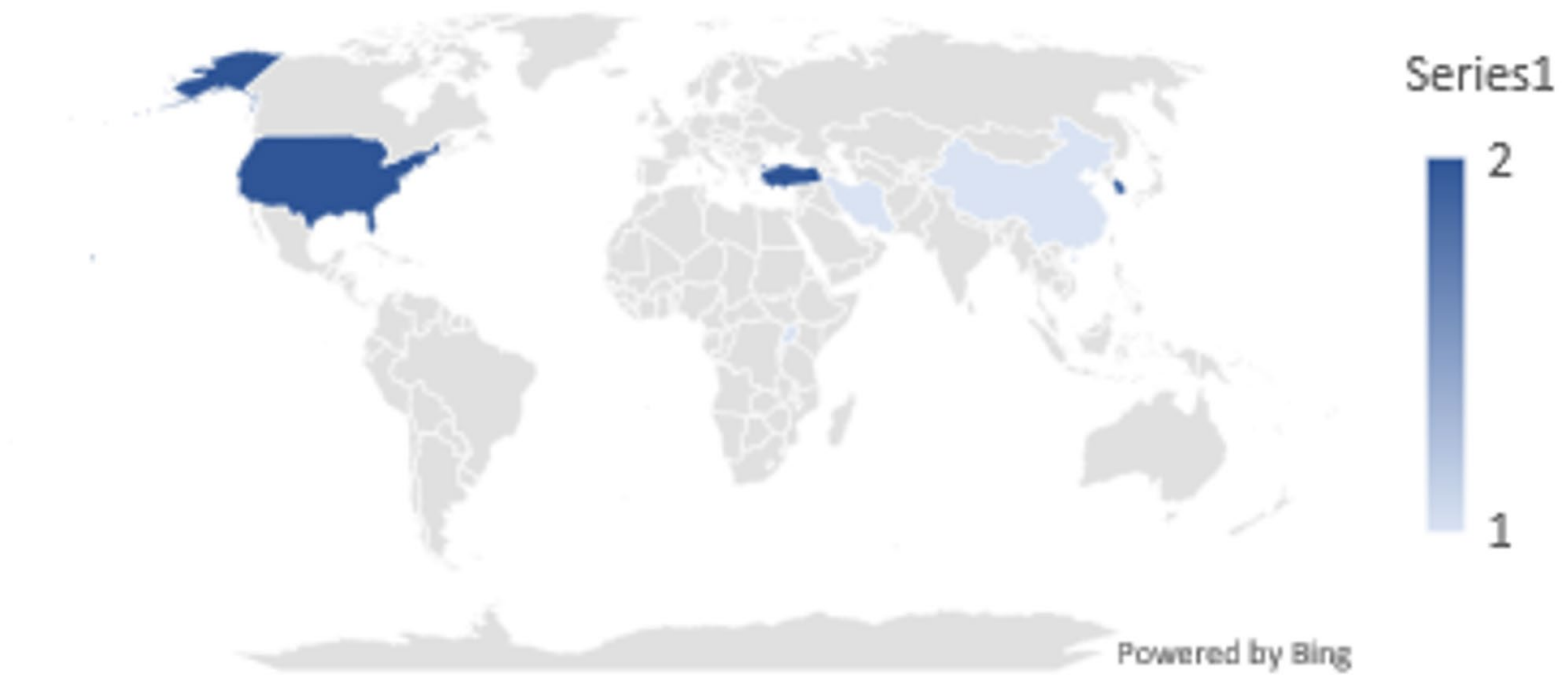
Distribution of included studies across countries

The included nine studies [10, 33, 34, 37–42] were published between 2011 and 2025, and were conducted across different geographical settings, including North America [38, 40], Asia [10, 37, 39, 41], Europe [33, 34] and Africa [42]. The distribution of studies across these regions is depicted in Fig. 2. Of the nine studies, eight employed a quantitative [10, 33, 34, 37–41] and one adopted a qualitative approach [42]. Though all studies were conducted among family members or close relatives of cancer patients, the definition of caregivers varied considerably across the included studies. Some studies included individuals most involved in caregiving, identified as primary or main caregivers [10, 37, 39, 40], indicating active caregiving. Others were family members who accompanied patients to the hospital or visited or stayed with them during hospitalisation [38, 41, 42], and a few included individuals who were co-resident with the patient, without explicit confirmation of their caregiving role [33, 34]. Breast cancer screening was most commonly examined [33, 34, 39–41], while a few studies focused on cervical cancer screening, either independently [42] or in combination with breast cancer screening [10, 37, 38]. Caregiving-related contextual variables were inconsistently reported across studies. Information on caregiving duration, living arrangements with the patient, inpatient or outpatient status, and caregiving burden was largely absent across studies, with caregiving burden assessed in only one study [10].

The characteristics of the included studies on factors influencing breast and cervical cancer screening, and on interventions that enhanced screening uptake, are presented in Tables 2 and 3, respectively.

**Table 2:** Summary of findings of factors influencing breast and cervical cancer screening

Sl. No.	Author, Year, country	Objective of the study	Study design	Setting	Type of cancer screening included	Inclusion criteria of participants (definition of caregiver)	Sample Size	Caregivers’ relationship to the patient	Diagnosis of the patient	Cancer stage of the patient	Duration of cancer diagnosis of the patient	Inpatient/Outpatient	Living with the patient or not	Caregiving burden (Assessed/not)	Factors
1	Origa M et al., 2025 Uganda	To understand knowledge of and facilitators and barriers to cervical cancer screening among attendants	Qualitative: Focus Group Discussion	Health facility	Cervical cancer screening	Female caregivers who were attendants of the cancer patients hospitalized at the health facility	40	Not specifically mentioned	Not specifically mentioned	Not specifically mentioned	Not specifically mentioned	Not specifically mentioned	Not specifically mentioned	No	Poor awareness Fear Stigma Cultural belief Privacy concern Husband’s hesitancy Lack of availability of nearest centres
2	Lu N et al., 2024 China	To explore the relevant factors of breast cancer screening behaviour in the FDRs of breast cancer patients based on the social cognitive the ory.	Quantitative: Cross sectional survey	Health facility	Breast cancer screening	First-degree relatives who were caregivers or visited the cancer patient in the hospital	430	Daughter: 54.2% Mother: 31.6% Sister: 14.2%	CA Breast	Not specifically reported	Less than 1 year: 61.6% More than 1 year: 38.4%	Not specifically mentioned	Not specifically reported	No	Self-efficacy Goal setting Positive and negative outcome expectations Social support
3	Takeuchi E et al., 2020 USA	To investigate the extent to which caregivers’ FCR predicted their cancer screening behaviors years after their relatives’ initial cancer diagnosis.	Quantitative: longitudinal study	Not specifically mentioned	Breast cancer screening	Individuals nominated by cancer survivors as their primary caregivers, including family members or family-like individuals (e.g., close friends) who provided consistent help throughout the cancer experience.	813 Female caregivers – 545	Spouse: 71% Parent: 4.3% Child: 14.1% Others: 10.3%	Not specifically reported	Not specifically reported	Not specifically reported		Not specifically reported	No	Age Ethnicity/Race Education Fear of Cancer Recurrence
4	Lin J et al., 2016 USA	To determine the incidence of cancer screening in caregivers of patients undergoing radiation oncology To identify barriers to and deficiencies in screening.	Quantitative: survey	Health facility	Breast and cervical cancer screening	Caregivers who accompanied the patient to radiation oncology consultation visits	209 Female caregivers − 146	Spouse: 43.5% Immediate family: 44% First- or second-degree relative: 4% Friend: 7% Employee or used by agency in charge of patient:1%	CA Brain: 4% CA Breast: 15% CA Head and Neck: 11% CA Lung: 11% CA Gastrointestinal: 20% CA Prostate: 13% CA Gynaecologic: 1% Others: 21% Don’t know: 1%	Not specifically reported	Not specifically reported		Not specifically reported	No	**Cervical cancer screening**: Area of residence (urban/rural) **Breast cancer screening**: Age Lack of awareness
5	Rha SY et al., 2015 Korea	To describe the caregiving burden and health-promoting behaviors of family caregivers of cancer patients To determine the relationship between caregiving burden and health-promoting behaviors among these caregivers.	Quantitative: Cross sectional survey	Health facility	Breast and cervical cancer screening	Individuals who are the main caregivers of the patients, family caregivers who are the most responsible for the care of cancer patients	227 Female caregivers − 183	Spouse: 49.3% Children: 35.2% Relatives: 15.4%	CA lung: 20.8% CA Colorectal: 19.9% CA Stomach: 15.9% CA breast: 10.6% Others: 32.8%	Stage 1: 4.6% Stage 2: 12.4% Stage 3: 17.9% Stage 4: 64.2%	Less than 12 months: 61.4% 12–23 months: 13% More than 24 months: 25.6%	Inpatient: 53.1% Outpatient: 49.1%	Yes: 69.6% No: 30.4%	Yes	Caregiving for a cancer patient
6	Koca D et al., 2013 Turkey	To evaluate the changes developing in the attitudes and behaviors toward prevention and cancer screening in the relatives of lung cancer patients.	Quantitative: survey	Not specifically mentioned (caregivers of patients who are under treatment)	Breast cancer screening	Any parent, sibling, child, or spouse who had been living with the patient for at least one year	246 Female caregivers − 86	Children: 35.7% Spouse: 35.4% Sibling: 14.3% Mother: 7.7% Father: 6.9%	Lung cancer	Stage 1: 23.2% Stage 2: 3.3% Stage 3: 22.4% Stage 4: 51.2%		Not specifically mentioned	All participants lived with the patient for at-least 1 year	No	Timepoint when the caregiver learnt about the cancer diagnos
7	Koca D et al., 2013 Turkey	Changes in the attitudes and behavior of relatives of breast cancer patients concerning cancer prevention and screening after diagnosis in a loved one were evaluated	Quantitative: survey	Not specifically mentioned (caregivers of patients who are under treatment)	Breast cancer Screening	First-degree relatives of the patients (mothers, sisters, and daughters) and to those who shared a home with the patient at least one year.	171	Mother: 28.1% Daughter: 34.5% Sister: 37.4%	Breast cancer	Stage 1: 13.5% Stage 2: 16.4% Stage 3: 12.9% Stage 4: 57.3%		Not specifically mentioned	All participants lived with the patient for at-least 1 year	No	Relationship to the patient Education Timepoint when the caregiver learnt about the cancer diagnosis
8	Son KY et al., 2011 Korea	To compare the status of lifestyle behavior and use of preventive services, such as screening tests for chronic disease and cancer screening, in spousal caregivers of cancer patients and controls	Quantitative: cross sectional	Health facility	Breast and cervical cancer screening	Spouses (who were assumed to be the primary caregivers) who accompanied the cancer patients to the hospital	200 (100 caregivers and 100 patients) 63- female caregivers	Spousal caregivers	CA Stomach: 12% CA lung: 22% CA Colorectal: 18% CA breast: 14% CA Lymphoma: 8% Others: 26%	Localized: 11% Regional: 32% Distant: 44%	Less than 1 year: 49% 1 year to less than 2 years: 19% 2 years to less than 3 years: 13% More than 3 years: 19%	Not specifically mentioned	Not specifically mentioned – but all were spousal caregivers	No	Being caregiver (spousal) to a cancer patient
9	Nasiriani K et al., 2017 Iran	To determine caregiver patients’ knowledge of the risk factors for breast cancer and practice of breast cancer screening among women with a positive family history of breast cancer. To assess the effect of telephone counseling and education on mammography screening.	Quantitative: community based trial (experimental design)	Not specifically mentioned (Data was collected through telephonic interviews)	Breast cancer screening	first-degree relative caregivers (mother, sister or daughter or any other) of breast cancer patients admitted in the hospital	90 (45- intervention group, 45- control group)	Mother: 22.2% Sister: 44.4% Daughter: 22.2% Others: 11.1%	Breast cancer	Not specifically mentioned	Not specifically mentioned	Not specifically mentioned	Not specifically mentioned	No	Lack of knowledge Belief Time Fear Forgetfulness Doctor recommendation Cost

**Table 3:** Summary of findings of intervention enhancing breast cancer screening

Sl. No.	Study	Study Design	Geographic location	Population	Sample Size	Age	Objective	Type of intervention	Comparator	Follow-up	Mode of intervention delivery	Frequency of intervention	Duration of intervention	Description of the intervention
1	Nasiriani K et al., 2017	Experimental design and a community-based trial	Iran	first-degree relative caregivers (mother, sister or daughter or any other) of breast cancer patients	90 (45- intervention group, 45- control group)	40 years and above	To assess the effect of telephone counseling and education on mammography screening.	Telephonic counselling and education	No intervention (control group received intervention after study period)	3 months	Nurse counsellor delivered the intervention	3 phone calls during the intervention period	Each call lasted for 60–120 min	information about the basics of breast cancer screening (breast self exam, clinical breast examination and mammography, time period, intervals, and screening location).

### Factors influencing the uptake of breast and/or cervical cancer screening among informal cancer caregivers

The included studies reported a range of factors influencing breast and/or cervical cancer screening. The factors were mapped across the domains of the “DOST (Determinants Of Screening upTake)” model [43]. Most reported factors were clustered within the individual-level domains, with fewer in the social, structural, and health system domains.

### Individual non-modifiable factors

#### Sociodemographic profile

Certain sociodemographic factors played an important role in the uptake of cancer screening among informal cancer caregivers. Higher breast cancer screening was reported among older caregivers [38, 40] white caregivers [40], and those who had higher education (college graduates) [33, 40]. Non-compliance with recommended cervical cancer screening was higher among urban caregivers than their rural counterparts [38].

#### Social identity

Social identity, as defined by the DOST model, is the “role in family or community as a social or financial provider and decision maker.” Caregiving status and relationship to the cancer patient were the social identity-related factors influencing cancer screening among informal cancer caregivers. Some included studies identified that caregivers had better uptake of both breast and cervical cancer screening than non-caregivers [10, 37]. Compared to other female relatives, sisters were more likely to undergo breast cancer screening [33].

### Knowledge and awareness

Insufficient knowledge of screening was a key barrier for both breast [38, 39] and cervical cancer screening [38]. Caregivers who were informed of their relative’s cancer diagnosis, after a period of time, underwent breast cancer screening compared to those who were informed at the time of diagnosis [33, 34].

### Perceived factors

Fear was consistently observed as a perceived factor across studies. Fear of cancer diagnosis hindered both breast [38, 39, 41] and cervical cancer screening [38]. Fear related to pain, social judgment, and spousal separation was reported as a barrier to cervical cancer screening [42]. Fear of cancer recurrence (FCR) of the survivors was associated with breast cancer screening among caregivers. Caregivers with moderate levels of FCR were more likely to maintain age-appropriate breast cancer screening, and high FCR levels led to inappropriate or excessive screening, particularly among younger caregivers [40].

Other perceived barriers, including stigma of being known as a cancer patient, privacy concerns, and cultural beliefs, hindered cervical cancer screening [42]. While forgetfulness, negative outcome expectations and the low perceived need hindered breast cancer screening [39], positive outcome expectations, goal setting, and perceived self-efficacy enhanced breast cancer screening among informal cancer caregivers [41].

### Structural and health system factors

While limited time and high screening cost emerged as the structural barriers to breast cancer screening [39], lack of nearby screening centre and the long distance to the centres were the structural barriers to cervical cancer screening [42]. Additionally, the lack of prescription by doctors emerged as a critical health-system-related barrier for breast cancer screening [39].

### Social factors

Social factors included the husband’s hesitancy, which discouraged caregivers from undergoing cervical cancer screening [42], and support from friends and family, which enhanced their breast cancer screening [41].

### Intervention that enhanced the uptake of breast and/or cervical cancer screening among informal cancer caregivers

Only one study was identified that enhanced the uptake of breast cancer screening in informal cancer caregivers. A community-based trial conducted in 2017 in Iran evaluated the effectiveness of telephonic counselling and education by a trained nurse on mammography screening uptake among informal cancer caregivers. A total of 90 informal caregivers, comprising 45 in the intervention group and 45 in the control group, were included. The intervention group received telephone counselling and education three times (once a month for three months) on breast cancer screening practices, timing, and locations of screening services. The control group received no intervention during the study period. Mammography screening uptake increased from 13.3% at baseline to 77.8% following the intervention (*P* < 0.001), while no significant change was observed in the control group [39]. No interventions were identified that specifically targeted informal cancer caregivers to enhance their uptake of cervical cancer screening. The scarcity of interventions targeting informal cancer caregivers indicates a limited research attention to this population in cancer screening and prevention research.

**Fig. 3:**
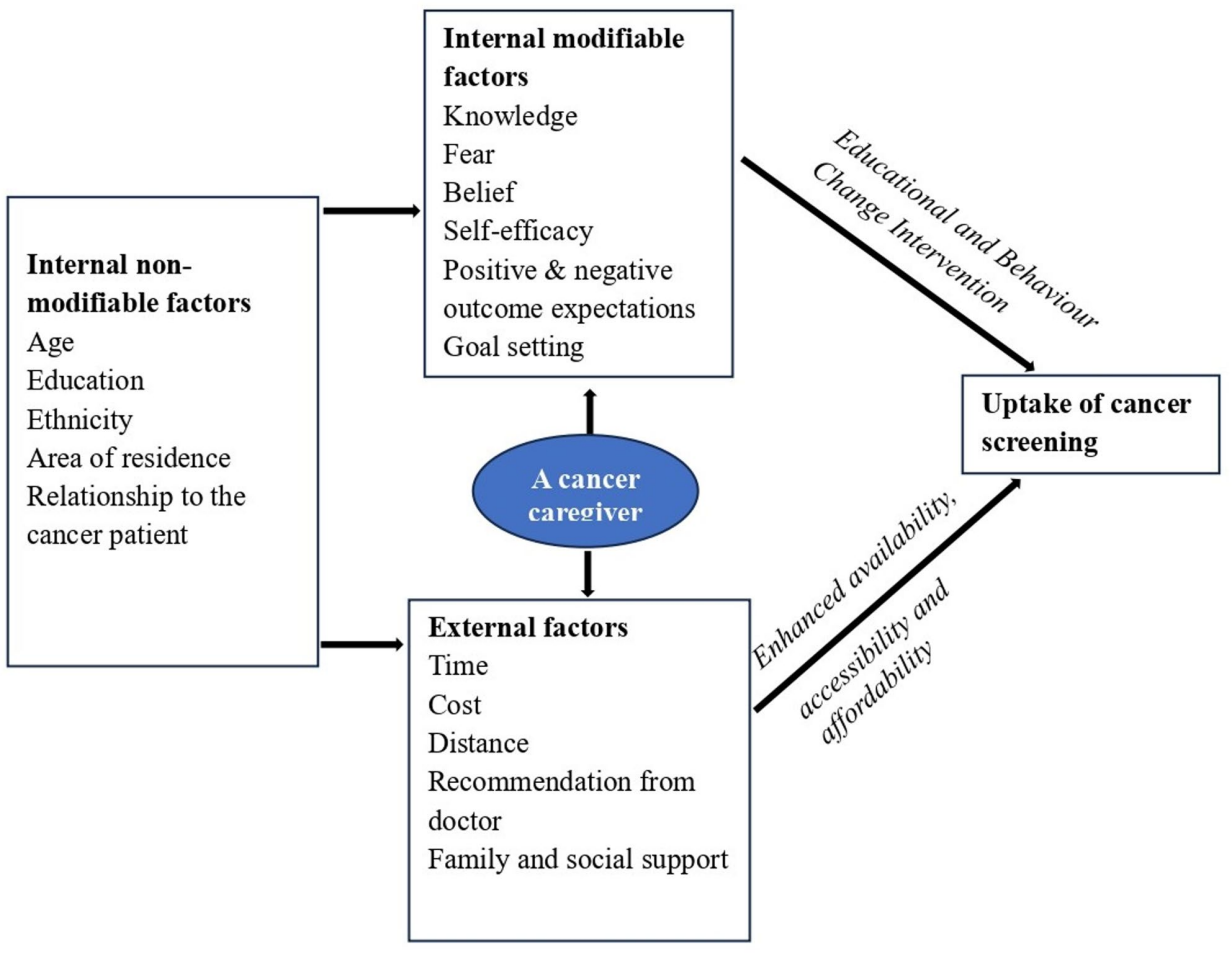
Factors influencing cancer screening among women who are informal cancer caregivers

Based on the findings from the included studies, the authors developed a conceptual framework (Figure 3) inductively. This framework is intended as a summary of the findings, providing a comprehensive understanding of the non-modifiable, modifiable, and external factors that contribute to cancer screening, and it also suggests strategies to address those factors/barriers, which may help guide future research.

## Discussion

### Findings directly supported by the review evidence

To the best of our knowledge, this is the first scoping review on breast and cervical cancer screening among “informal cancer caregivers.” The findings indicate that most reported factors, including socio-demographic characteristics, awareness, perceived factors, structural and health system barriers, and social influences, largely mirror those commonly observed among the general female population [4, 44–47]. However, it cannot be concluded that caregiving has no unique influences on the screening behaviour, as the potential caregiving-specific contextual factors, such as caregivers’ relationships with the patient, the patient’s diagnosis and cancer stage, duration since diagnosis, caregivers’ cohabitation with the patient, and caregiving burden, have not been adequately examined in the existing literature, highlighting a research gap and not the absence of caregiving-related influences. This gap in the existing literature restricts our ability to understand how the cancer caregiving context may influence screening behaviour, including whether caregiving intensifies existing barriers or adds new ones to undergo breast and cervical cancer screening.

### Broader insights from related literature

Some included studies suggested higher breast and cervical cancer screening uptake among caregivers than non-caregivers [10, 37]. The cancer diagnosis of a family member can act as a “cue to action” towards cancer preventive health behaviour, which may result in enhanced cancer screening uptake among informal cancer caregivers [48]. However, informational, emotional, structural and social factors hindered screening.

The insufficient knowledge as a barrier to cancer screening among informal cancer caregivers identified in this review indicates that information gaps persist even among individuals closely involved in the cancer care continuum. Although caregivers are often engaged in information-seeking behaviour, they usually seek information about the patient’s disease, treatment, and side effects [49] rather than their personal risk or need for screening. This suggests that informational exposure within the caregiving context does not always equip caregivers with the knowledge required for self-protective behaviour such as cancer screening. Targeted strategies are therefore required to provide caregivers with screening-specific information and to support their engagement in breast and cervical cancer screening.

While fear of cancer diagnosis acted as a barrier for both breast and cervical cancer in many caregivers, a moderate level of FCR of the cancer survivors’ motivated informal caregivers to undergo breast cancer screening [40]. This indicates how witnessing cancer suffering can influence cancer screening behaviour positively and negatively. Caregivers who witness the firsthand experience of cancer may develop an awareness of the importance of early cancer detection [37]. However, many will be in a state of confusion, where they recognise the importance but delay or avoid undergoing cancer screening. In these contexts, awareness alone will be insufficient to motivate them to undergo screening. Supportive counselling and positively framed messages can help transform this ambiguity into preventive action rather than avoidance of screening. Moreover, informal cancer caregivers experience caregiving-related emotional responses, including anxiety and depression [50]. These conditions may influence their preventive health behaviour, but current evidence is limited and requires further investigation.

### Authors’ proposed implications for policy, practice and research

Primary care and oncology physicians are uniquely positioned to identify the informal cancer caregivers during their routine clinical encounters with cancer patients. Integrating brief education and counselling during these encounters can be an influential strategy to enhance cancer screening within the existing cancer care pathway. Additionally, opportunistic cancer screening within the oncology setting, where the family member receives treatment, can be a promising approach to address accessibility-related barriers to screening faced by informal cancer caregivers.

Presently, the interventions targeting informal cancer caregivers to enhance their breast and/or cervical cancer screening are notably scarce. The paucity of caregiver-focused interventions suggests that the informal cancer caregivers have not been systematically prioritised in cancer screening and prevention research. While interventions targeting the general female population may raise caregivers’ awareness of cancer screening, they might be insufficient to motivate them to undergo cancer screening unless their caregiving-related contextual barriers are addressed. Hence, targeted approaches that address their unique barriers and concerns are needed to enhance their breast and cervical cancer screening behaviour. However, the current body of literature does not adequately examine caregiving-related contextual factors influencing breast and cervical cancer screening, thereby limiting understanding of such barriers.

Hence, future quantitative research should specifically examine how different caregiving-related contextual factors, such as caregivers’ relationships with the patient, the patient’s diagnosis and cancer stage, duration since diagnosis, caregivers’ cohabitation with the patient, and caregiving burden, influence the uptake of breast and cervical cancer screening and such studies should clearly define who constitute an “informal cancer caregiver”, considering criteria such as the relationship to the patient, the nature and extent of caregiving tasks, and the duration and extent of caregiving involvement.

Qualitative research is equally necessary to uncover the various barriers, challenges, and facilitators for cancer screening. Findings from such studies can inform the development of targeted strategies to enhance cancer screening and prevention among this largely unexplored group. In response, the authors are conducting research among informal cancer caregivers to understand their engagement in cervical cancer screening and to enhance screening uptake in a low- and middle-income setting.

### Strengths and limitations

This scoping review has several strengths. First, it addresses an underexplored yet important population – informal caregivers of cancer patients, a group that is often overlooked in cancer prevention and screening research, despite their close involvement in the cancer care continuum. Second, the review used focused research questions, which enabled us to comprehensively capture the existing evidence on both factors influencing breast and/or cervical cancer screening and interventions that enhance it in this minimally explored population. Third, the review followed a rigorous, systematic methodology guided by established scoping review frameworks. Fourth, the review employed a comprehensive search strategy across multiple databases and grey literature sources, thereby enhancing the breadth of evidence and reducing the risk of missing relevant studies. Fifth, the use of the DOST model to categorise the influencing factors adds conceptual clarity to the findings. Finally, the review identified critical gaps in the literature, particularly the inadequate exploration of caregiving-specific contextual factors and the lack of caregiver-targeted interventions.

However, the following limitations should be acknowledged when interpreting the findings. First, the findings are based on a limited number of studies; therefore, the review’s findings cannot be generalised to the entire caregiver population. Second, due to the limited number of primary studies conducted in this population, we used broadened inclusion criteria that allowed studies conducted among those identified as primary caregivers, those who accompany, stay with, or visit the patient at the hospital, or those who co-reside with them at home. Given this heterogeneity in definitions, our findings must be interpreted with caution, as factors observed among primary caregivers may differ from those among those who accompany the patient in the hospital or co-reside with the patient at home. Due to the limited number of studies and limited evidence on the factors and interventions in the included studies, we could not compare the observed findings across these different caregiver groups. Third, the limited examination of caregiving-related contextual factors in the included studies limited our ability to conclude how caregiving influences breast and/or cervical cancer screening. Lastly, we included only studies published in English, which may have resulted in the exclusion of relevant studies published in other languages.

## Conclusion

This scoping review indicates that global evidence on the factors influencing and strategies to enhance breast and/or cervical cancer among informal cancer caregivers is limited. However, the existing evidence indicates that informational, emotional and structural barriers hinder their cancer screening. Caregiving-specific contextual factors and their association with breast and cervical cancer screening practice are rarely examined in the existing studies, and the definition of “informal caregiver” varied across the included studies, limiting the ability to design context-sensitive interventions to enhance cancer screening for this population. A simple low-cost intervention, such as telephonic counselling, significantly enhanced their breast cancer screening uptake. Future research should clearly define who constitutes an “informal cancer caregiver”, examine caregiving-context-specific factors and evaluate targeted interventions, particularly for cervical cancer screening, to inform equitable cancer screening and prevention efforts for this overlooked population.

## Supplementary Information

Supplementary Material 1.

Supplementary Material 2.

## Data Availability

All data generated or analysed during this study are included in this published article [and its supplementary information files].
