# Supplementary material for: Caring for the caregivers: breast and cervical cancer screening among informal caregivers of cancer patients – a scoping review
Source: BMC Prim Care. 2026 May 4;27:245. doi: 10.1186/s12875-026-03333-2 (PMC13321855; doi:10.1186/s12875-026-03333-2)
Supplement: Supplementary file 2 — Supplementary Material 2. [file 12875_2026_3333_MOESM2_ESM.docx]

**PubMed Search string**

((("caregiver*"[MeSH Terms] OR "caregive*"[Text Word] OR "Carers"[Text Word] OR "family caregiver*"[Text Word] OR "spouse caregivers"[Text Word] OR "informal caregivers"[Text Word] OR "Women caregivers"[All Fields] OR Caregivers [Text Word])) AND (("breast neoplasm*"[MeSH Terms] OR Breast Neoplasm*[Text Word] OR Breast Tumor*[Text Word] OR Breast Cancer[Text Word] OR Cancer of Breast[Text Word] OR Malignant Neoplasm of Breast[Text Word] OR Breast Malignant Neoplasm*[Text Word] OR Malignant Tumor of Breast[Text Word] OR Mammary Cancer[Text Word] OR Human Mammary Neoplasm*[Text Word] OR Breast Carcinoma*[Text Word] OR Human Mammary Carcinoma*[Text Word] OR "uterine cervical neoplasm*"[MeSH Terms] OR cervical cancer*[Text Word] OR Uterine Cervical Neoplasm*[Text Word] OR Cervix Neoplasm*[Text Word] OR Cancer of the Uterine Cervix[Text Word] OR Cancer of Cervix[Text Word] OR Cervix Cancer*[Text Word] OR Uterine Cervical Cancer*[Text Word] OR Cervical Cancer*[Text Word]))) AND (("mass screening"[MeSH Terms] OR "early detection of cancer"[MeSH Terms] OR "screening"[Text Word] OR "screening uptake"[All Fields] OR "Cancer screening" [All fields]))

Limited to English language studies
